# Conjunctivitis: A Systematic Review

**DOI:** 10.18502/jovr.v15i3.7456

**Published:** 2020-07-29

**Authors:** Amir A. Azari, Amir Arabi

**Affiliations:** ^1^Ophthalmic Research Center, Research Institute for Ophthalmology and Vision Science, Shahid Beheshti University of Medical Sciences, Tehran, Iran; ^2^Department of Ophthalmology, Torfeh Medical Center, Shahid Beheshti University of Medical Sciences, Tehran, Iran

**Keywords:** Allergic, Bacterial, Conjunctivitis, COVID-19, Coronavirus, Viral, Toxic

## Abstract

Conjunctivitis is a commonly encountered condition in ophthalmology clinics throughout the world. In the management of suspected cases of conjunctivitis, alarming signs for more serious intraocular conditions, such as severe pain, decreased vision, and painful pupillary reaction, must be considered. Additionally, a thorough medical and ophthalmic history should be obtained and a thorough physical examination should be done in patients with atypical findings and chronic course. Concurrent physical exam findings with relevant history may reveal the presence of a systemic condition with involvement of the conjunctiva. Viral conjunctivitis remains to be the most common overall cause of conjunctivitis. Bacterial conjunctivitis is encountered less frequently and it is the second most common cause of infectious conjunctivitis. Allergic conjunctivitis is encountered in nearly half of the population and the findings include itching, mucoid discharge, chemosis, and eyelid edema. Long-term usage of eye drops with preservatives in a patient with conjunctival irritation and discharge points to the toxic conjunctivitis as the underlying etiology. Effective management of conjunctivitis includes timely diagnosis, appropriate differentiation of the various etiologies, and appropriate treatment.

##  INTRODUCTION

Conjunctivitis is characterized by inflammation and swelling of the conjunctival tissue, accompanied by engorgement of the blood vessels, ocular discharge, and pain. Many subjects are affected with conjunctivitis worldwide, and it is one of the most frequent reasons for office visits to general medical and ophthalmology clinics. More than 80% of all acute cases of conjunctivitis are reported to be diagnosed by non-ophthalmologists including internists, family medicine physicians, pediatricians, and nurse practitioners.^[1]^ This imposes a great economic burden to the healthcare system and occupies a great proportion of the office visits in many medical specialties. It is estimated that the cost of treating bacterial conjunctivitis is $857 million annually in the United States alone.^[2]^

It has been reported that nearly 60% of all patients with acute conjunctivitis receive antibiotic eye drops; and the vast majority receive their prescription from a non-ophthalmologist physician. For example, 68% of patients who visited a physician at an emergency room received antibiotic eye drops while this figure dropped to 36% for those who saw an ophthalmologist.^[1]^ Interestingly, patients from a higher socioeconomic status were more likely to receive and fill a prescription for their conjunctivitis.^[1]^

There are several ways to categorize conjunctivitis; it may be classified based on etiology, chronicity, severity, and extend of involvement of the surrounding tissue. The etiology of conjunctivitis may be infectious or non-infectious. Viral conjunctivitis followed by bacterial conjunctivitis is the most common cause of infectious conjunctivitis, while allergic and toxin-induced conjunctivitis are among the most common non-infectious etiologies. In terms of chronicity, conjunctivitis may be divided into acute with rapid onset and duration of four weeks or less, subacute, and chronic with duration longer than four weeks.^[3]^ Furthermore, conjunctivitis may be labeled as severe when the affected individuals are extremely symptomatic and there is an abundance of mucopurulent discharge. Conjunctivitis may be associated with the involvement of the surrounding tissue such as the eyelid margins and cornea in blepharoconjunctivitis and viral keratoconjunctivitis, respectively.

Additionally, conjunctivitis may be associated with systemic conditions, including immune-related diseases [e.g., Reiter's, Stevens-Johnson syndrome (SJS), and keratoconjunctivitis sicca in rheumatoid arthritis], nutritional deprivation (vitamin A deficiency), and congenital metabolic syndromes (Richner-Hanhart syndrome and porphyria)^[4,5]^ (Table 1).

**Table 1:** Guideline to help differentiate the major etiologies in conjunctivitis


**Clinical history and exam findings**	**Most probable etiologies**
**Alarming signs and symptoms**	
Decreased vision, severe pain, painful pupillary reaction, anisocoria, orbital signs	Uveitis, scleritis, keratitis, glaucoma, orbital, or parasellar pathology
**Chronicity**	
Sudden onset, lasting less than four weeks	Infectious conjunctivitis, allergic conjunctivitis, acute systemic reactions (SJS/TEN)
Insidious onset, chronic course	Conjunctivitis associated with systemic diseases, toxic conjunctivitis, allergic conjunctivitis
Recurrent course	Allergic conjunctivitis, conjunctivitis associated with systemic diseases
**Associated symptoms**	
Skin lesions, arthropathy, genito-perineal involvement, oropharyngeal lesions	Conjunctivitis associated with systemic diseases, infectious diseases
**Drug history**	
Long-term eye drop usage	Toxic conjunctivitis, allergic conjunctivitis
Recent initiation of a systemic medication	Acute systemic reactions (SJS/TEN)
SJS, Stevens-Johnson syndrome; TEN, toxic epidermal necrolysis	

**Table 2:** Selected non-conjunctivitis etiologies of red eye


**Differential diagnosis**	**Symptoms**	**Exam findings**
Dry eyes	Burning and FB sensation. Symptoms are usually transient, worse with reading or watching TV due to decreased blinking. Symptoms are worse in dry, cold, and windy environments due to increased evaporation	Bilateral redness, superficial punctate keratopathy, meibomian glands dysfunction, decreased tear break-up time, small tear meniscus
Blepharitis	Similar to dry eyes	Redness greater at the margins of eyelids, inflammation, telangiectasia, and crust around eyelashes
Pterygium	Recurrent ocular redness	Visible conjunctival extension over the cornea
Hordeolum, chalazion	Eyelid pain and swelling	Palpable eyelid mass, may be tender or not
Anterior segment tumors	Variable	Variable
Corneal abrasion, keratitis, corneal foreign body	FB sensation, relevant history including contact lens usage and occupational exposure	Corneal epithelial defects, corneal infiltration, corneal FB
Contact lens overwear	Relevant history	Corneal epithelial defect
Subconjunctival hemorrhage	Ocular redness	Blood under conjunctiva
Scleritis	Decreased vision, moderate to severe pain	Redness, bluish scleral hue
Iritis	Photophobia, pain, blurred vision. Symptoms are usually bilateral	Decreased vision, poorly reacting pupils, constant eye pain radiating to temple and brow. Redness, severe photophobia, presence of inflammatory cells in the anterior chamber
Angle closure glaucoma	Headaches, nausea, vomiting, ocular pain, decreased vision, light sensitivity, and seeing haloes around lights. Symptoms are usually unilateral.	Firm eye upon palpation, ocular redness with limbal injection. Appearance of a hazy/steamy cornea, moderately dilated pupils that are unreactive to light.
Carotid cavernous fistula	Chronic red eye, may have a history of head trauma	Dilated tortuous vessels (corkscrew vessels), bruits upon auscultation with a stethoscope
Endophthalmitis	Severe pain, photophobia, may have a history of eye surgery or ocular trauma	Redness, puss in the anterior chamber and photophobia
Cellulitis	Pain, double vision, and fullness	Redness and swelling of lids, may have restriction of the eye movements, may have a history of preceding sinusitis (usually ethmoiditis)
FB, foreign body; TV, television			

It is extremely important to differentiate conjunctivitis from other causes of “red eye” associated with severe sight- or life-threatening consequences such as acute angle closure glaucoma, uveitis, endophthalmitis, carotid-cavernous fistula, cellulitis, and anterior segment tumors.

##  METHODS

The scientific literature published as of February 2020 was thoroughly reviewed by searching PubMed, the ISI web of knowledge database, and the Cochrane library using relevant keywords. The following keywords were used: "bacterial conjunctivitis", "viral conjunctivitis," "allergic conjunctivitis", "treatment of bacterial conjunctivitis", and "treatment of viral conjunctivitis". No language restriction was applied.

Articles published between March 2013 and February 2020 were screened and those that provided the best evidence-based information were included in this review. A total of 167 articles were finally included. The first study was published in 1964 and the last study was published in 2020.

##  History and clinical examination

### How to diagnose conjunctivitis

Conjunctival injection or “red eye” is a shared presentation for many ophthalmic diseases, and it accounts for up to 1% of all primary care office visits.^[6]^ The clinicians, whether ophthalmologist or not, must be aware that “red eye” may be the presenting sign for serious eye conditions such as uveitis, keratitis, or scleritis, or it may be secondary to more benign conditions that are limited just to the conjunctival tissue (e.g., conjunctivitis or subconjunctival hemorrhage). Traditionally, it was believed that more harmful ophthalmic disorders are associated with disturbances in vision, disabling pain, and photophobia.^[6]^ However, in a recent large meta-analysis,^[6]^ anisocoria and mild photophobia were significantly associated with “serious eye conditions”; the presence of these two signs could discover 59% of cases of “serious eye conditions”, including anterior uveitis and keratitis. Table 2 provides a summary of the main etiologies of “red eye” and their clinical characteristics.

### How to distinguish infectious conjunctivitis from non-infectious conjunctivitis 

Obtaining history from patients who present with conjunctivitis is crucial in order to arrive at the correct diagnosis. A focused ocular history should include the following: onset and duration of symptoms; laterality; impairment of vision; presence of itching; contact lens wear history; presence of fellow travelers such as recent upper respiratory infection, sinusitis, and lymphadenopathy; previous episodes of conjunctivitis; systemic allergies and medication; and history of exposure to chemical agents.

The presence of constitutional signs such as fever, malaise, fatigue, and contact with individuals with conjunctivitis helps to further narrow down the differential diagnosis. Physical examination, including checking for palpable lymph nodes, especially in the periauricular and submandibular areas, is of great importance. Ophthalmic examination should be performed to determine the type of discharge. Closer examination using a slit-lamp biomicroscope to evaluate the ocular surface structures including the palpebral conjunctiva for the presence of pseudomembranes, symblepharon, papilla or follicles, and the corneal tissue for the presence of opacities and infiltrates is absolutely essential.

Some of the clinical signs and symptoms that are used to help diagnose infectious conjunctivitis include the following: eye discharge, conjunctival injection, presence of red eye(s), eyelashes being stuck together in the morning, grittiness of the eye(s), eyelid or conjunctival edema, and history of contact with individuals with conjunctivitis.^[7]^

Allergic conjunctivitis may be underdiagnosed and undertreated.^[8]^ It is presented with itching, chemosis, and redness in the absence of any significant corneal involvement.^[9]^ The degree of conjunctival swelling is often out of proportion to conjunctival hyperemia. The main findings in vernal keratoconjunctivitis (VKC) are the presence of giant papillae in the superior tarsal conjunctiva accompanied by severe itching,^[10]^ while the presence of conjunctival scar and anterior subcapsular cataract supports the diagnosis of atopic keratoconjunctivitis (AKC).^[11]^

Another similar condition, chronic toxic conjunctivitis, may present with watery discharge, an initial papillary conjunctival reaction followed by a follicular reaction, punctate epithelial erosion of the cornea, and eyelid dermatitis.^[12,13,14]^

### How to distinguish bacterial conjunctivitis from viral conjunctivitis 

Predicting the underlying etiology of conjunctivitis based on the presenting signs and symptoms may often result in an inaccurate diagnosis. In one study, centers with expertise in ocular surface disease had an accuracy rate of only 48% in making the correct diagnosis of adenoviral conjunctivitis.^[15]^ Several other studies demonstrated that bacterial pathogens are only isolated in 50% of cases of suspected bacterial conjunctivitis.^[16]^ In addition, one study reported that up to 52% of presumed cases of viral conjunctivitis were culture-positive for bacteria.^[15]^

Traditionally, the following associations between the clinical history and the etiology of conjunctivitis were believed to be true; these principles were presented in many textbooks and were used to select patients in many clinical trials.^[17]^ For example, according to the major text books in ophthalmology (e.g., Krachmer, Duane, and Kanski), involvement of one eye followed by the involvement of the second eye within 24–48 hours is indicative of bacterial infection, while if the second eye becomes infected after 48 hours with an accompanying enlarged periauricular lymph node, a viral etiology should be considered. According to the same textbooks, a papillary conjunctival reaction or pseudomembranous conjunctivitis strongly suggests a bacterial origin for conjunctivitis while follicular conjunctival reaction is more likely to indicate a viral etiology.

There are many other associations between the etiology of conjunctivitis and symptoms that are thought to be true, but lack strong clinical evidence. For example, association between lack of itching and bacterial conjunctivitis have come under scrutiny in the recent years. Other associations that once thought to be true but lack evidence include: recent upper respiratory tract infection and lymphadenopathy in favor of viral conjunctivitis; sinusitis, fever, malaise, and fatigue in association with bacterial conjunctivitis; and previous history of conjunctivitis with bilateral involvement of the eyes in favor of viral and allergic but not bacterial conjunctivitis.

A meta-analysis in 2003 failed to find any clinical studies correlating the signs and symptoms of conjunctivitis with its underlying etiology.^[17]^ Following the above meta-analysis, a prospective study was conducted and found that combination of three signs, bilateral mattering of the eyelids, lack of itching, and no previous history of conjunctivitis were strong predictors of bacterial conjunctivitis.^[18]^ Having both eyes matter and their eyelashes adhere together in the morning was a stronger predictor for positive bacterial culture, and either itching or a previous episode of conjunctivitis made a positive bacterial culture less likely. In addition, types of the discharge (purulent, mucus, or watery) or other symptoms were not specific to any particular class of conjunctivitis.

A more recent meta-analysis, which analyzed the clinical data of 622 patients from three clinical trials,^[19]^ found that patients with purulent discharge or mild to moderate red eye were less likely to benefit from topical antibiotics; this finding reiterates lack of meaningful correlation between signs and symptoms and the underlying etiology in most cases of conjunctivitis. Another recent study in 2013 found a strong likelihood of positive bacterial culture results in patients with the “gluing of the eyelids” upon waking up in the morning, and the age above 50 at presentation.^[20]^

###  How do laboratory findings help us?

Clinicians may collect discharge samples from eyes with conjunctivitis and send them for microbiological evaluation. Conjunctival cultures are generally reserved for cases of suspected infectious neonatal conjunctivitis, recurrent conjunctivitis, conjunctivitis recalcitrant to therapy, conjunctivitis presenting with severe purulent discharge, and cases suspicious for gonococcal or chlamydial infection.^[21]^ Swabs from the discharge are better to be taken before the initiation of antimicrobial therapy. The swabs are then plated in various growth mediums in the laboratory for obtaining cultures. Sabouraud agar plates are used to identify fungus, and it should be utilized in patients with chronic blepharitis and those who are immunocompromised. Anaerobic culture plates may also be helpful, especially in patients with a history of previous surgery or trauma.^[22]^ If antimicrobial therapy has already been started, they should be stopped 48 hours prior to obtaining cultures. In a five-year review of 138 pediatric ocular surface infections, the most common organisms were coagulase-negative *staphylococci*, followed by *Pseudomonas aeruginosa* and *Staphylococcus aureus*.^[23]^

Nucleic acid amplification techniques, requiring special swabs, may be used in diagnosing viral infections, where a multitude of polymerase chain reaction (PCR) tests for detection of viruses are available.

Although primary studies from in-office rapid antigen testing for adenoviruses report 89% sensitivity and up to 94% specificity,^[21]^ the results of more recent studies point toward a high specificity but only moderate sensitivity ranging from 39.5% to 50%.^[24]^ Accordingly, it may be suggested that negative Adeno-Plus test results should be confirmed by real-time PCR owing to its suboptimal sensitivity.

For those suspected of having allergic conjunctivitis, skin scratch test or intradermal injection of common allergens, and assays for detecting elevated *in vitro* levels of specific serum IgE may be used; however, the diagnosis of allergic conjunctivitis remains a clinical one.

##  Viral conjunctivitis

Viral conjunctivitis is the most common overall cause of infectious conjunctivitis, and it is usually secondary to inoculation of the ocular surface with the adenoviruses.^[25,26]^ Less frequently, other viruses may be the underlying etiology in viral conjunctivitis; amongst them, herpes simplex virus (HSV), varicella zoster virus (VZV), and enterovirus have been the subject of investigation.^[27]^

### Adenoviral conjunctivitis

As the leading cause of infectious conjunctivitis worldwide, up to 90% of viral conjunctivitis cases are caused by adenoviruses.^[28]^ Recent advances in genome sequencing of human adenoviruses (HAdV) have identified over 72 unique HAdV genotypes classified into seven different species (HAdV-A through HAdV-G), with HAdV-D species having the most members and the strongest association with viral conjunctivitis.^[29,30]^

Perhaps the most common form of infection by the adenoviruses in children is pharyngoconjunctival fever (PCF) caused by HAdV types 3, 4, and 7.^[31,32,33]^ This condition is usually characterized by the presence of fever, pharyngitis, periauricular lymphadenopathy, and acute follicular conjunctivitis. Additional ocular surface findings include edema, hyperemia, and petechial hemorrhages of the conjunctiva as a result of interaction between pro-inflammatory cytokines and conjunctival vasculature.^[32]^ This condition is self-limited, often resolving spontaneously in two–three weeks without any treatment.

**Figure 1:**
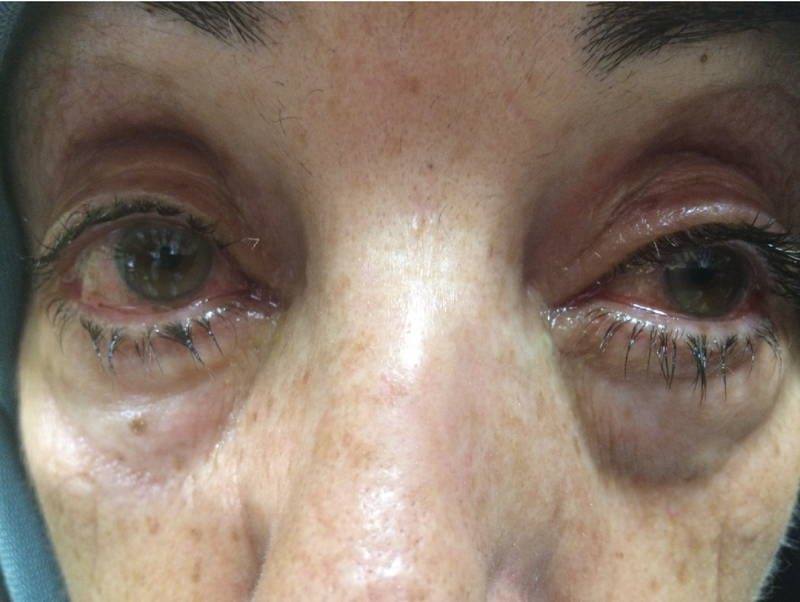
Adenoviral conjunctivitis presenting as bilateral watery eyes.

**Figure 2:**
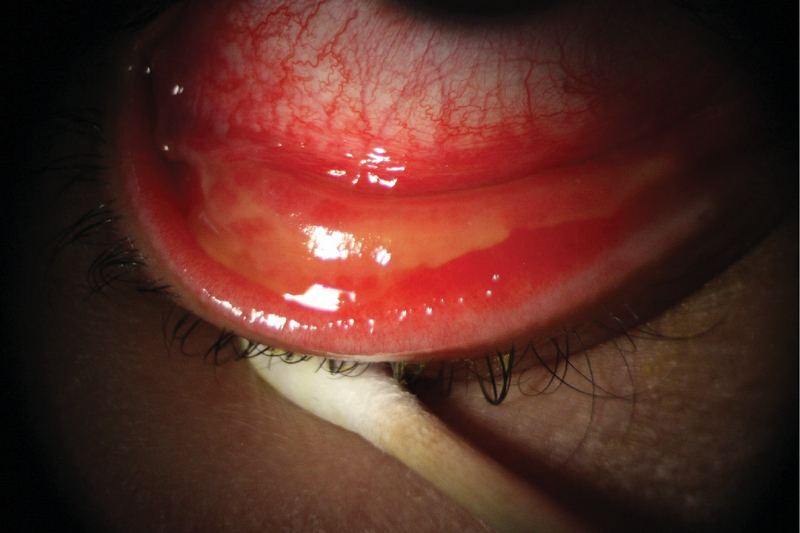
Pseudomembrane formation in a patient with adenoviral conjunctivitis.

The most severe ocular manifestation of adenoviral infection is the epidemic keratoconjunctivitis (EKC); this condition affects both the conjunctiva and cornea, leaving behind long-lasting and permanent ocular surface changes and visual disturbances. Ocular manifestations of EKC include conjunctival discharge, follicular conjunctivitis, corneal subepithelial infiltrates (SEI), corneal scarring, development of conjunctival membranes and pseudomembranes, and symblepharon formation (Figures 1 and 2).

**Figure 3:**
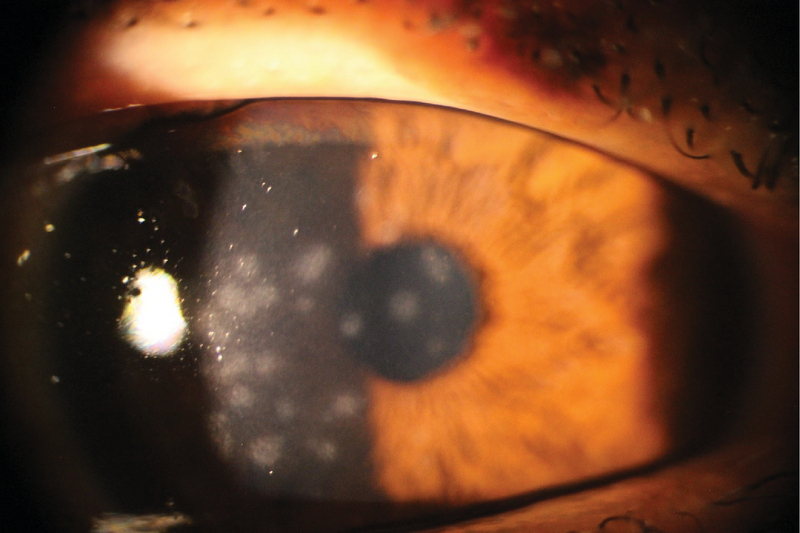
Subepithelial infiltrations in a patient with adenoviral conjunctivitis.

Classically, serotypes 8, 19, 37, and less frequently serotype 4 were believed to be associated with EKC, but more recently, HAdV-D53 and HAdV-D54 have been identified in several outbreaks and are thought to be responsible for the majority of EKC cases.^[30]^

Pseudomembranes, which are sheets of fibrin-rich exudates without blood or lymphatic vessels, may be encountered in the tarsal conjunctiva of the EKC patients.^[35]^ Depending on the intensity of inflammation, true conjunctival membranes may also form in EKC. True membranes, once form, can lead to the development of subepithelial fibrosis and symblepharon; additionally, they tend to bleed severely upon removal.^[36]^

Cornea is another tissue that may become adversely affected in EKC. Replication of the virus in the corneal epithelium may cause superficial punctate keratopathy, followed by focal areas of epithelial opacities.^[37]^ Focal SEI in the anterior stroma of the cornea appears approximately 7–10 days following the initial involvement of the eyes with EKC^[38]^ (Figure 3). These opacities may persist for years, and they may be associated with visual disturbance, photophobia, and astigmatism. The incidence of SEI formation in EKC has been reported to vary from 49.1 to 80%.^[39]^ An immunologic reaction to the replicating adenoviruses in anterior stromal keratocytes is hypothesized to be the underlying mechanism for the formation of SEIs. The observation that these opacities recur following discontinuation of steroids supports the hypothesis.^[40]^

Adenovirus conjunctivitis is very contagious and it may be transmitted up to 50% of the time according to some reports.^[41,42]^ The virus may spread through contaminated fingers, medical devices, contaminated water at the swimming pools, or by sharing of personal items; as many as 46% of individuals with viral conjunctivitis had positive viral culture grown from their hands according to one study.^[43]^ The adenovirus is a very hardy organism, and it is reported to be resistant to 70% isopropyl alcohol and 3% hydrogen peroxide.^[44]^ The American Academy of Ophthalmology recommends using a 1:10 dilute bleach solution (sodium hypochlorite) to disinfect the office equipment and instruments against common infectious agents encountered in eye care clinics including the adenoviruses.^[45]^

Due to the highly contagious nature of viral conjunctivitis, frequent hand washing, meticulous disinfection of medical instruments, and isolation of conjunctivitis patients from the rest in the healthcare provider's office has been recommended.^[46]^ The incubation period for the adenovirus is approximately 5–12 days, while the infected individuals can transmit the disease for up to 14 days from the time they are infected.^[41]^

There is no single effective treatment modality for viral conjunctivitis; however, use of frequent artificial tears, antihistamines containing eye drops, or cold-compresses seem to alleviate many of the clinical symptoms that are associated with this condition.^[47,48]^ Topical and oral antiviral medications do not appear to be useful.^[47,48]^ In addition, antibiotic eye drops do not play a role in treating viral conjunctivitis and may even obscure the clinical picture by inducing ocular surface toxicity.^[15,16]^ Other concerns with using antibiotic drops include increased bacterial resistance and the possibility of spreading the disease to the contralateral eye by cross-contamination through the infected bottles.^[42]^

Membranes or pseudomembranes may be peeled at the slit-lamp by using a pair of jeweler forceps or cotton swab after anesthetizing the ocular surface. This is done to alleviate patient discomfort and prevent future scar formation.

Monotherapy against viral conjunctivitis with Povidone-iodine 2% have been investigated in a pilot study. The authors discovered that topical administration of Povidone-iodine 2% four times a day for one week led to complete resolution of the disease in three-quarters of the eyes.^[49]^

The American Academy of Ophthalmology suggests that topical corticosteroids play an important role in the treatment of conjunctivitis, but they should be used judiciously and with caution in selected cases.^[47]^ Indications for steroid usage in viral conjunctivitis are membrane formation and sub-epithelial infiltration associated with severe photophobia and decreased vision. Prolonging the duration of adenoviral conjunctivitis, exacerbation of HSV keratitis, and an increase in intraocular pressure are the main adverse effects of indiscriminate use of topical corticosteroids.

Prolongation of viral shedding following monotherapy with corticosteroids has been reported;^[50]^ however, combination therapies with corticosteroids and anti-infective agents (i.e., antibiotics) have proven to be effective in treating viral and bacterial conjunctivitis.^[51,52]^

Ophthalmic formulations of PVP-I/dexamethasone are widely investigated. PVP-I 0.4%/dexamethasone 0.1% suspension, PVP-I 1.0%/dexamethasone 0.1%, and PVP-I 0.6%/dexamethasone 0.1% have been used, and the results suggest that the combination therapies reduce patient symptoms and eradicate the virus effectively.^[50,53,54,55]^

Ongoing phase 3, randomized, double-masked, controlled studies will further clarify the efficacy and safety of combined PVP-I/dexamethasone in adenoviral conjunctivitis (ClinicalTrials.gov identifiers: NCT0299855441 and NCT0299854142) and bacterial conjunctivitis (ClinicalTrials.gov identifiers: NCT03004924).

Use of 1 and 2% cyclosporine-A (CsA) eye drops have been advocated for the treatment of SEIs, and it has been demonstrated to be effective in improving patient symptoms and reducing the amounts of infiltrates.^[30,56]^ However, Jeng et al suggested that it might be difficult to wean patients completely off CsA once they have started it; in their study, when CsA was stopped, SEIs returned, necessitating reinstitution of the CsA eye drops.^[57]^ This finding is in contrast with the Reinhard's pilot study, where no recurrence was observed after discontinuation of the CsA drops.^[58]^ In a small study consisting of 39 patients, administration of 1% cyclosporine-A (four times a day) during the acute phase of viral conjunctivitis and continuing it thereafter for 21 days lowered the incidence of corneal opacities significantly.^[59]^ A case-controlled double-blinded randomized clinical trial is needed to investigate the effectiveness of cyclosporine-A and to formulate an ideal tapering regiment for this medication.

The use of topical tacrolimus eye drops has also been investigated for the treatment of SEIs secondary to adenoviral keratoconjunctivitis. When tacrolimus eye drops or ointments were used for an average of six months, a significant reduction in the size and numbers of SEIs was observed in 60% of the cases, while in 31.76% of the eyes, SEIs were eliminated after one year.^[60]^ There was also a statistically significant improvement in the visual acuity of the patients with the use of topical tacrolimus.

### Herpetic conjunctivitis

It is estimated that 1.3–4.8% of all cases of acute conjunctivitis are caused by HSV infection.^[61,62,63]^ HSV often causes a unilateral follicular conjunctivitis, which may be accompanied by a thin watery discharge and associated vesicular lesions on the skin of the eyelids. Treatment consists of topical antiviral agents, including ganciclovir, idoxuridine, vidarabine, and trifluridine. The purpose of the treatment is to reduce virus shedding and the chance of the development of keratitis.

Ocular involvement with herpes zoster virus, especially when the first and second branches of the trigeminal nerve are involved, can lead to conjunctivitis in 41.1% of cases, eyelid lesions in 45.8%, uveitis in 38.2%, and corneal lesions such as SEIs, pseudodendrites, and nummular keratitis in another 19.1%.^[64,65]^

### Acute hemorrhagic conjunctivitis

Acute hemorrhagic conjunctivitis (AHC) is an extremely contagious form of viral conjunctivitis. It manifests by foreign body sensation, profuse tearing, eyelid edema, dilatation of conjunctival vessels, chemosis, and subconjunctival hemorrhage. In a small proportion of patients, fever, fatigue, and leg pain may ensue. Two picornaviruses, namely enterovirus 70 (EV70) and coxsackievirus A24 variant (CA24v), as well as certain subtypes of adenoviruses are believed to be the responsible pathogens.^[66,67,68]^ Like the other forms of conjunctivitis, AHC is also believed to be transmitted primarily by hand-to-eye-to-hand contact and infected fomites.^[69]^ The condition is self-limited and the symptoms diminish gradually during the first week of infection and completely resolves after 10–14 days.^[69]^ Medical intervention aims primarily at controlling the large outbreaks as well as instituting preventative measures to protect the vulnerable groups, such as children, elderly, pregnant women, and immunocompromised individuals, by encouraging frequent handwashing and reducing contact with the affected individuals.^[68]^

### Miscellaneous viral conjunctivitis

Infection with Molluscum contagiosum (MC) is characterized by multiple umblicated and papular skin lesions caused by Pox-2 virus. Skin-to-skin contact and sexual intercourse are the main routes of transmission. Shedding of the viral proteins from the eyelid lesions into the tear film leads to chronic follicular conjunctival reaction, punctate keratopathy, and subepithelial pannus. Rarely, primary MC lesions are found in the conjunctiva.^[70]^

Ebola hemorrhagic fever is a fatal disease caused by the species of ebolavirus. Conjunctival injection, subconjunctival hemorrhage, and tearing have been reported in the affected individuals.^[71]^ Conjunctival injection, which is often bilateral and present in up to 58% of cases, has been identified in both the acute and late stages of this disease and may play an important role in the early diagnosis of this potentially deadly condition.^[72]^ While human-to-human transmission through bodily fluids can spread the infection, the natural reservoir is thought to be the fruit bat.^[73]^

Coronaviruses include a broad family of viruses that normally affect animals, although some strains can spread from animals to humans.^[74]^ The most recently isolated strain of coronavirus, “2019-nCoV”, has made the headlines since it was first recognized in December 2019 in China. COVID-19 has been reported to cause fever, cough, shortness of breath, and even death.^[75,76]^ Some reports have suggested that this virus can cause conjunctivitis and be transmitted via the conjunctival secretions of the infected individuals.^[76]^ All healthcare professionals including the ophthalmologists should be vigilant in approaching patients with conjunctivitis and respiratory symptoms, especially if they report a recent history of travel to high risk regions.^[76]^

##  Bacterial conjunctivitis

While in adults, bacterial conjunctivitis is less common than viral conjunctivitis, in children, it is encountered more frequently.^[77]^ Bacterial conjunctivitis can result from either a direct contact with infected individuals or from abnormal proliferation of the native conjunctival flora.^[78]^ Contaminated fingers,^[41]^ oculogenital spread,^[47]^ and contaminated fomites^[79]^ are common routes of transmission. In addition, certain conditions such as compromised tear production, disruption of the natural epithelial barrier, abnormality of adnexal structures, trauma, and immunosuppressed status increase the likelihood of contracting bacterial conjunctivitis.^[47]^

**Figure 4:**
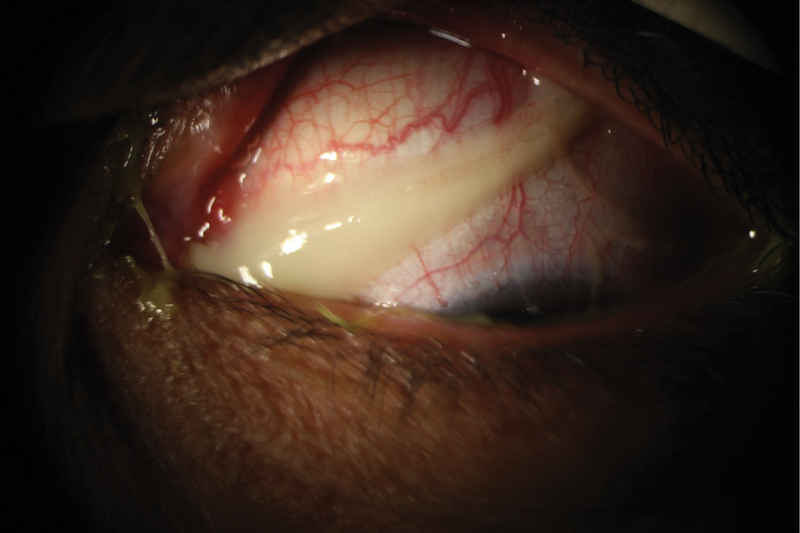
Thick purulent discharge in a patient with acute bacterial conjunctivitis.

Acute bacterial conjunctivitis is most often caused by *Staphylococcus* species, *Haemophilus influenza*, *Streptococcus* species, *Moraxella catarrhalis*, and gram-negative intestinal bacteria.^[80]^ In younger children, minor epidemics may occur secondary to *H. influenza* or *S. pneumonia*. Acute bacterial conjunctivitis manifests by foreign body sensation and increased ocular secretion in addition to moderate conjunctival hyperemia (Figure 4).

Several studies on bacterial conjunctivitis^[81,82]^ demonstrate that sticky eyelids and itching may be present in approximately 90% of the affected individuals; these findings are followed by the less frequently encountered signs and symptoms such as purulent secretion and ocular burning. *H. influenza* conjunctivitis may be associated with acute otitis media and upper respiratory tract infection.^[80]^

In more than 60% of cases, spontaneous cure occurs within one–two weeks,^[83]^ and serious complications are extremely rare.^[84]^ However, presence of a large population of bacteria on the conjunctiva exposes the patient to a higher risk of keratitis, particularly in conditions associated with corneal epithelial defects, such as dry eye.^[80]^

Although topical antibiotics reduce the duration of the disease, no difference in the outcome is seen between the treatment and placebo groups. In a meta-analysis,^[81]^, consisting of 3,673 patients from 11 randomized clinical trials, antibiotic treatment increased the rate of clinical improvement by 10% compared to placebo. Both “2 to 5” and “6 to 10” day regiments were included in this analysis. Although, highly virulent bacteria can potentially inflict serious damage to the ocular surface and the eye,^[78]^, no sight-threatening complications were reported in any of the placebo groups in the aforementioned meta-analysis.^[85]^

All broad-spectrum antibiotic eye drops seem to be effective in treating bacterial conjunctivitis and it is unlikely that there is a significant difference among various antibiotics in achieving clinical cure. Factors that influence antibiotic choice are local availability, patient allergies, resistance patterns, and cost.

From a large systematic review, it was concluded that topical antibiotics were more effective in achieving clinical and microbial cure when patients had positive bacterial cultures.^[21]^ However, no significant difference has been reported in clinical cure rate when different frequencies of the antibiotics were administered.^[86,87]^ Due to lengthening the course of the illness and potentiating the infection, topical steroids should be avoided^[47]^ (Table 3).

**Table 3:** Ophthalmic drug therapies for acute bacterial conjunctivitis.


**Antibiotic agents**	**Treatment**
**Aminoglycosides**	
Gentamicin	Ointment: 4 ×/d for 1 wk Solution: 1-2 drops 4 ×/d for 1 wk
Tobramycin	Ointment: 3 ×/d for 1 wk
**Fluoroquinolones**	
Besifloxacin	1 drop 3 ×/d for 1 wk
Ciprofloxacin	Ointment: 3 ×/d for 1 wk Solution: 1-2 drops 4 ×/d for 1 wk
Gatifloxacin	3 ×/d for 1 week
Levofloxacin	1-2 drops 4 ×/d for 1 wk
Moxifloxacin	3 ×/d for 1 wk
Ofloxacin	1-2 drops 4 ×/d for 1 wk
**Macrolides**	
Azithromycin	2 ×/d for 2 d; then 1 drop daily for 5 d
Erythromycin	4 ×/d for 1 wk
**Sulfonamides**	
Sulfacetamide	Ointment: 4 ×/d and at bedtime for 1 wk Solution: 1-2 drops every 2-3 h for 1 wk
**Combination** **drops**	
Trimethoprim/polymyxin B	1 or 2 drops 4 ×/d for 1 wk
	
	

**Figure 5:**
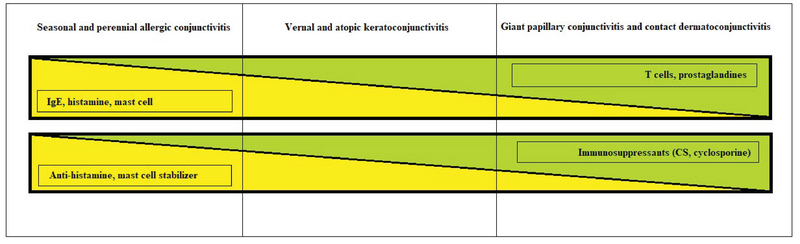
Spectrum of allergic conjunctivitis. CS, corticosteroid

**Figure 6:**
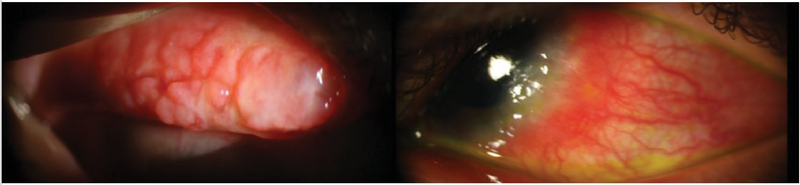
Cobblestone appearance of large conjunctival papillae in a patient with VKC (left). Limbal VKC with Horner-Trantas dots in another patient (right).

**Figure 7:**
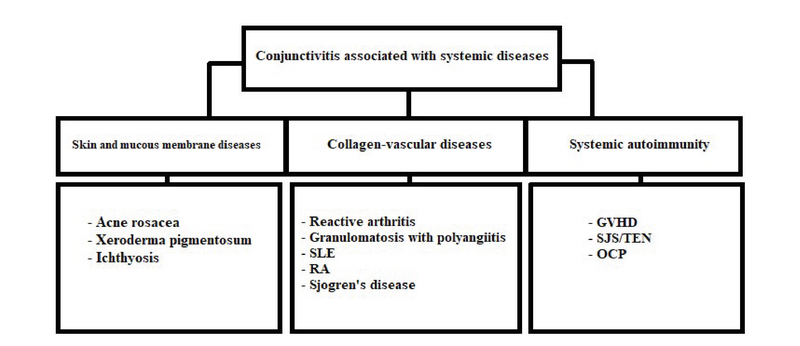
Some systemic and dermatological conditions associated with conjunctivitis.

**Figure 8:**
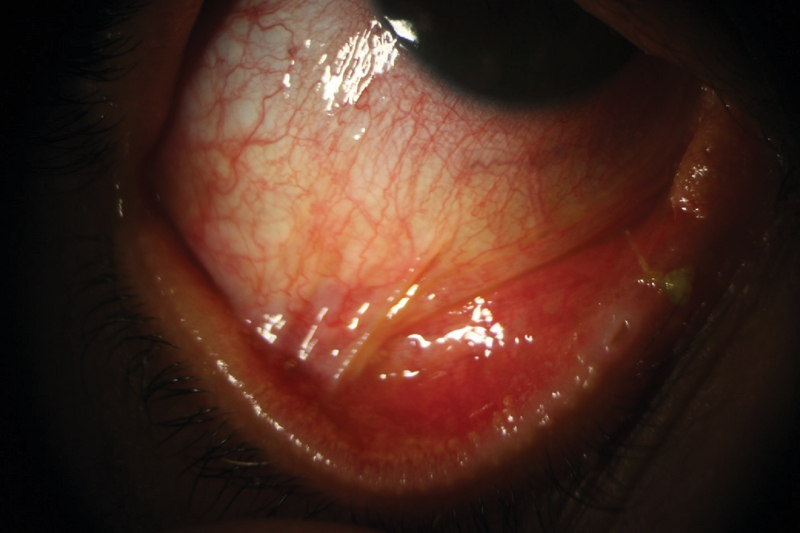
Symblepharon formation in a patient with ocular cicatricial pemphigoid.

### Methicillin-resistant S. aureus conjunctivitis

The term methicillin-resistant *S. aureus* (MRSA) refers to *Staphylococcus aureus* species that are resistant to methicillin antibiotic; however, nowadays the term is used to describe resistance to all β-lactam antimicrobials.^[88]^ Growing in prevalence, 3–64% of all ocular *Staphylococcus* conjunctival infections are MRSA conjunctivitis.^[89]^ Suspected cases need to be treated with fortified vancomycin eye drops or ointments.^[90]^ Culture-directed administration of antimicrobials, effective dosing, considering the local resistance patterns, and appropriate antiseptic strategies should be applied to restrict the spread of MRSA conjunctivitis.^[91]^

### Chlamydial conjunctivitis 

*Chlamydia trachomatis* may cause a variety of ocular surface infections including trachoma, neonatal conjunctivitis, and inclusion conjunctivitis. Serotype D-K are causative agents for neonatal conjunctivitis and adult inclusion conjunctivitis, while trachoma is caused by serotypes A, B, Ba, and C.^[92]^

Inclusion conjunctivitis is reported to cause 1.8–5.6% of all cases of acute conjunctivitis,^[61,62,93]^ where the majority of cases are unilateral and have concurrent genital infection.^[94]^ Patients often present with mild mucopurulent discharge and follicular conjunctivitis persisting for weeks to months.^[77]^ Up to 54% of men and 74% of women are reported to have simultaneous genital infection.^[95]^ The disease is frequently acquired via oculogenital spread.^[47]^ Treatment with systemic antibiotics such as oral azithromycin and doxycycline is efficacious, while addition of topical antibiotics is not beneficial. Treatment of sexual partners and looking for the evidence of coinfection with gonorrhea must be instituted.

As the leading cause of infectious blindness in the world, trachoma affects 40 million individuals worldwide; this infection is prevalent in areas with poor hygiene. Although mucopurulent discharge is the initial presenting sign, in the later stages, scarring of the eyelids, conjunctiva, and cornea may lead to loss of vision. A single dose of oral azithromycin (20 mg/kg) in addition to oral tetracycline or erythromycin for three weeks is very effective. Patients may also be treated with topical antibiotic ointments, such as tetracycline and erythromycin, for six weeks.^[96,97]^

In newborns, chlamydia can cause conjunctivitis following passage through an infected birth canal. The acute phase, which typically begins between days 5 and 14 following vaginal delivery, is characterized by purulent discharge, erythema and edema of the eyelids and conjunctiva.^[98]^ More prevalent than gonococcal conjunctivitis (GC), neonatal conjunctivitis secondary to *C. trachomatis* is considered the most frequent infectious cause of neonatal conjunctivitis worldwide.^[98,99,100]^

Although the chlamydial conjunctivitis has a mild course, scarring of the cornea and/or conjunctiva have been reported in untreated cases.^[101]^ It is important to note that up to 20% of the neonates who are exposed to chlamydia may develop pneumonia; in these, 50% demonstrate a previous history of conjunctivitis.^[102]^

A recent meta-analysis supports the superiority of traditional treatment with systemic erythromycin at 50 mg/kg per day (given in four divided doses for two weeks), in comparison to topical antibiotic therapy alone.^[103]^ A recent study evaluating the efficacy of azithromycin in neonatal chlamydial conjunctivitis^[104]^ demonstrated superiority of erythromycin over azithromycin; however, risk of pyloric stenosis related to the use of erythromycin may reduce its clinical use in neonates in the future.^[103]^ Additionally, less-frequent dose of azithromycin may improve compliance.^[105]^

### Gonococcal conjunctivitis (GC)

Typically viewed as a condition affecting the neonates, GC, however, affects other age groups as well.^[106]^ *Neisseria gonorrhoeae* is a common cause of hyperacute conjunctivitis in neonates and sexually active adults.^[78]^ Ocular infection with *N. gonorrhea* is associated with a high prevalence of corneal perforation.^[80]^ GC should be considered as the causative agent in neonates who present with conjunctivitis in days 2 to 5 after delivery.^[106]^ In both neonatal and non-neonatal populations, eye exam may reveal conjunctival injection and chemosis along with copious mucopurulent discharge; a tender globe with periauricular lymphadenopathy may also be associated with this type of conjunctivitis.^[106]^

The suggested treatment for neonates include single dose of ceftriaxone (25 to 50 mg/kg), or cefotaxime (100 mg/kg IV or IM), in addition to hourly saline irrigation of the ocular surface.^[106,107,108]^ Non-neonates can be treated with combination of 1 gm of IM ceftriaxone given in a single dose and 1 gm of oral azithromycin (which is used to treat the frequently encountered chlamydial coinfection). Irrigation of the ocular surface with saline solution is not necessary in adults.^[106]^

##  Allergic conjunctivitis

Ocular allergy can affect the entire ocular surface including conjunctiva, eyelids, and cornea. According to the immunological mechanism responsible for the final clinical picture, Leonardi et al have classified ocular allergic conditions into three main categories:^[109]^ IgE-mediated reactions, including seasonal allergic conjunctivitis (SAC) and perennial allergic conjunctivitis (PAC); combined IgE and non-IgE-mediated reactions, including VKC and AKC; and non-IgE-mediated reactions, including giant papillary conjunctivitis (GPC) and contact dermatoconjunctivitis (CDC) (Figure 5).

### Seasonal allergic conjunctivitis (SAC) and perennial allergic conjunctivitis (PAC)

SAC and PAC are considered as the most prevalent allergic ocular conditions, affecting 15–20% of the population.^[110]^ The pathogenesis is predominantly an IgE-mediated hypersensitivity reaction, and allergen-specific IgE antibodies are found in almost all cases of SAC and PAC.^[111]^ Activation of mast cells contributes to increased levels of histamine, prostaglandins, and leukotrienes in the tear film. This phase, which is known as the early response phase, clinically lasts 20–30 min.^[8]^

SAC, also known as hay fever conjunctivitis, is seen in all age groups. The ocular manifestations occur predominantly during the spring and summer months when pollens from the trees and plants are released into the air. PAC on the other hand can occur throughout the year with exposure to more common allergens such as animal hair, mites, and feathers.^[112]^ Clinical signs and symptoms are similar in SAC and PAC, and include itching and burning of the eyes, tearing, and rhinorrhea. Corneal involvement is rarely seen.^[9]^

### Vernal keratoconjunctivitis (VKC)

VKC is known as the disease of young males who live in warmer climates.^[113,114]^ Although VKC is frequently diagnosed in children, adults can also be affected with this condition.^[115]^ A mixture of IgE and non-IgE reaction in response to nonspecific stimuli, such as wind, dust, and sunlight is often elucidated in this condition. Accordingly, skin tests and serum IgE antibody tests to well-known allergens are generally negative.^[116]^ Both clinical and histological findings support the concomitant role of T-helper 2 and IgE in the pathogenesis of VKC.^[8,117]^ Recently, IL-17 has been reported to be linked to VKC, where its serum levels can serve as a marker for the severity of the disease.^[118,119]^ High percentage of antinuclear antibodies (ANA) positivity and family history of autoimmune disorders in patients with VKC suggests a strong link between this condition and other autoimmune disorders including atopy.^[120,121]^

Typical seasonal patterns as well as perennial forms have been reported in patients affected with VKC.^[122]^ Presence of papillary hyperplasia is essential for the diagnosis of VKC, and its presence allows for the differentiation of VKC from other related entities such as SAC and PAC.^[123]^

Conjunctival injection, profuse tearing, severe itching, and photophobia are the main clinical signs and symptoms that are associated with VKC. There are three clinical forms of VKC that include limbal, palpebral, and mixed type.^[112]^ Limbal type is characterized by limbal papillary reaction and gelatinous thickening of the limbus; when the disease is active, Horner-Trantas dots are usually present at the superior limbal margins.^[112]^ The hallmark of the palpebral VKC is the presence of giant papillae, with consequent cobblestone appearance. The mixed type has the features of palpebral and limbal VKC simultaneously (Figure 6).

The corneal pathology that is seen in VKC is partly caused by the mechanical trauma from the tarsal conjunctival papillae and the inflammatory responses secondary to the release of cytokines. The inflammatory mediators are believed to be released by the eosinophils and mast cells that are infiltrated into the conjunctival tissue.^[124,125]^ In up to 6% of patients, corneal ulcers (i.e., shields ulcer) and plaques may develop, leading to the exacerbation of the clinical symptoms and worsening of the vision.^[126,127]^ These ulcers are usually found as oval lesions with elevated margins surrounding a chronic epithelial defect covered by eosinophilic and epithelial debris in the upper parts of the cornea.^[128]^ Keratoconus is another entity that is highly associated with VKC affecting nearly 15% of the patients with this condition.^[129]^

### Atopic keratoconjunctivitis (AKC)

AKC is characterized by chronic allergic disease of the eyelid, cornea, and conjunctiva. It is considered the ocular component of atopic dermatitis (AD), and roughly 95% of the patients with AKC have concomitant AD;^[8,11]^ however, less than half of patients with AD have involvement of their ocular tissue.^[130]^ Many cytokines are released from the epithelial cells of the conjunctiva as well as the inflammatory cells that have infiltrated the conjunctival tissues in AKC. This causes constant remodeling of the ocular surface connective tissue leading to mucus metaplasia, scar formation, and corneal neovascularization.^[131]^

AKC is typically diagnosed in the second and third decades of life, although scattered cases are seen in the early childhood as well as in the fifth decade of life.^[132]^ Age of the onset, duration of the disease, and clinical presentations may help clinicians to distinguish this condition from VKC.^[132]^

Clinical manifestation of AKC includes epiphora, itching, redness, and decreased vision. Presentation is often bilateral; however, unilateral disease has been reported.^[133]^ The eyelid skin may be edematous with a sandpaper-like texture. Conjunctival injection and chemosis range from mild to severe, and conjunctival scarring is common.^[11]^ Trantas dots and giant papillae may or may not be present. In contrast to VKC, AKC is associated with conjunctival fibrosis and corneal vascularization and opacities. An early cataract surgery is not uncommon in AKC patients, as this condition is associated with formation of “atopic cataracts” at a relatively young age. Shield-like cataracts, as well as nuclear, cortical and even posterior subcapsular cataracts may also occur. Nearly 50% of AKC patients test negative for common allergens.^[8]^

### Giant papillary conjunctivitis (GPC)

Similar to vernal conjunctivitis, GPC is characterized by papillary hypertrophy of the superior tarsal conjunctiva.^[134]^ Although GPC is primarily considered as a complication of contact lens usage, this condition has also been reported in association with corneal foreign bodies, filtering blebs, ocular prostheses, exposed sutures, limbal dermoids, and tissue adhesives.^[135,136,137]^ The classic signs of GPC consist of excessive mucous secretion associated with decreased contact lens tolerance.^[137]^ Mast cells and eosinophils may be found in the conjunctiva; however, there are no increases in the levels of IgE or histamines in the tears of patients with GPC.^[8]^

GPC can occur with both hydrogel and rigid contact lenses, and it has been reported with either hydroxyethyl methacrylate (HEMA), silicone polymers, or the new gas permeable polymers.^[134]^ However, it is less frequent with rigid contact lenses. Mechanical injuries due to contact lens wear and inflammatory reactions secondary to surface proteins of the lens can contribute to the chronic inflammatory damage of the ocular surface^[110,138]^ seen in this condition.

### Contact allergy

CDC is a classic example of type-IV delayed hypersensitivity reaction that occurs through interaction of antigens with T cells followed by release of cytokines.^[139]^ Low molecular weight allergens combine with host proteins to form the final allergens capable of exerting immune response. Some of the known allergens for CDC include poison ivy, poison oak, neomycin, nickel, latex, atropine and its derivatives.^[8]^ Primary sensitization phase describes the process through which memory T cells derive from resident T cells of the ocular tissue, while the following elicitation phase includes the interaction between these memory cells and allergens.^[8]^ IL-17-producing Th cells and regulatory T cells also play a role in the pathogenesis of CDC.^[140]^

Similar to AKC, contact allergy involves the conjunctiva, cornea, and eyelids. The condition may be associated with itching, lid swelling, follicular reaction, and even cicatrization in later stages of the disease. The corneal involvement may be in the form of punctate keratitis, pseudodendritic keratitis, and grayish stromal infiltrates.^[112,141]^

### Treatment

Avoidance of the allergens is the main stay of treatment for many forms of allergies including allergic conjunctivitis. Artificial tears provide a barrier function, dilute various allergens, and flush the ocular surface clean from many inflammatory mediators.

The treatment options for allergic conjunctivitis include lubricating eye drops, anti-histamines, and mast cell stabilizers.^[142,143]^ Many studies have demonstrated the superiority of topical antihistamines and mast cell stabilizers compared to placebo in alleviating the symptoms of allergic conjunctivitis; in addition, it has been demonstrated that antihistamines are more beneficial than mast cell stabilizers for providing short-term relief.^[144]^ Several eye drop preparations with dual action (antihistamine and mast cell-stabilizing effects) including olopatadine, ketotifen, azelastine, and epinastine have been introduced to market in the recent years. These agents can provide simultaneous histamine receptor antagonist effects, stabilize mast-cell membranes, and modify the action of eosinophils.^[145]^ Mast cell stabilizers require a loading period of several weeks, and therefore, they are better to be administered before the antigen exposure.

Oral antihistamines are commonly used for alleviating the ocular symptoms in patients with allergic conjunctivitis. Second generation antihistamines are preferred due to their fewer adverse systemic side effects.^[146]^ Unfortunately, oral antihistamines induce ocular drying, which can significantly worsen the symptoms of allergic conjunctivitis.^[147]^

Steroids should be used judiciously and only in selected cases. Topical and oral administration, in addition to supratarsal injections are often required if the condition is severe; unfortunately, any route of corticosteroid administration is associated with formation of cataracts and elevated intraocular pressure.^[112]^ Non-steroidal anti-inflammatory drugs such as ketorolac and diclofenac can also be added to the treatment regimen to provide additional benefits. Moreover, other steroid-sparing agents such as cyclosporine-A and tacrolimus are effective in treating severe and chronic forms of ocular allergies.

Allergen-specific immunotherapy, which has gained popularity in the recent years, works by inducing clinical tolerance to a specific allergen. This appears to be an effective treatment options for those with allergic rhinoconjunctivitis who demonstrate specific IgE antibodies.^[148]^ Traditionally, immunotherapy is performed via subcutaneous injections; however, sublingual immunotherapy (SLIT) has drawn the attention among allergists as an alternative. SLIT has been shown to effectively reduce the ocular and nasal signs and symptoms of allergic conjunctivitis, with a greater benefit toward improving the nasal symptoms.^[112]^

##  Conjunctivitis associated with systemic diseases

Conjunctivitis may be the initial presentation for many systemic diseases; therefore, a thorough history and systemic evaluation in selected cases may help in early diagnosis of many potentially disabling and even life-threatening conditions. A summary of systemic diseases associated with conjunctivitis is provided in Figure 7.

### Reactive arthritis

Conjunctivitis is one of the most common ocular manifestations of reactive arthritis; other associated ocular entities include uveitis, episcleritis, scleritis, and keratitis.^[149]^ Conjunctivitis in reactive arthritis entities manifests itself as conjunctival hyperemia with purulent discharge. Occurring in nearly one third of the patients, conjunctivitis is an essential component of the “Reiter's triad”.^[150]^ Conjunctivitis usually happens early in the course of reactive arthritis and it may even precede it in some instances; given its mild initial clinical presentation, it is often missed. The signs and symptoms usually abate within one to four weeks; however, in some cases, progression to more severe ocular surface problems may ensue.^[151]^

### Rosacea

Ocular surface may also be involved in the inflammatory course of ocular rosacea. The clinical findings include a follicular and papillary conjunctival reaction in association with interpalpebral conjunctival hyperemia. In addition, cicatrization of the conjunctival tissue, mimicking trachoma, may be seen in these patients. Conjunctival scarring secondary to entropion and trichiasis has been reported to occur in approximately 10% of the cases. Conjunctival granuloma, pinguecula, phlyctenule, and peripheral corneal infiltration and phlyctenule are amongst some of the other findings associated with ocular rosacea.^[152]^

### Graft-versus-host disease

Conjunctival involvement is rarely seen in acute graft-versus-host disease (GVHD); however, its presence indicates more severe systemic involvement and a poor prognosis. Conjunctival involvement in GVHD ranges from mild conjunctival injection to pseudomembranous and cicatrizing conjunctivitis.^[153,154]^ In acute GVHD, conjunctivitis is often ulcerative and manifests itself with numerous alternating episodes of conjunctival hemorrhage and exudative discharge. Sterile purulent discharge, pseudomembrane formation, and scarring are amongst the other findings in this condition.^[153]^ In the chronic form of GVHD, one-fourth to three-fourth of the patients suffer from dry eyes, where its severity correlates with the severity of GVHD.^[155]^ Frequently, keratoconjunctivitis sicca persists after remission of GVHD.^[156]^

Four stages of conjunctival GVHD have been described in the literature. Stage 1 is marked by simple conjunctival injection. Stage 2 is characterized by an exudative response, which may lead to conjunctival chemosis. Stage 3 is characterized by pseudomembrane formation; majority of the patients are diagnosed at this stage of the diseases. Stage 4 is manifested by scarring and cicatrization of the conjunctival tissue.^[153,156]^

### Ocular cicatricial pemphigoid

Ocular cicatricial pemphigoid is a rare condition. Patients are often in their fifth and sixth decades of life at presentation, and females are up to three times more frequently affected than males.^[157]^ Chronic inflammation, loss of conjunctival goblet cells along with an abnormal mucosal epithelial turn-over leads to desiccation of the ocular surface in this condition^[158]^ (Figure 8). Disruption of conjunctival immune network increases the risk of ocular surface infection.^[158]^ Recurrent infectious conjunctivitis and trichiasis may lead to keratinization of the surface epithelium.^[158]^ Definitive diagnosis requires direct immunofluorescence, where deposits of immunoglobulins and/or complements produce areas of linear hyperfluorescence at the epithelial basement membrane. Systemic immunosuppression along with frequent lubrication is often needed to adequately control this condition.

### Stevens-Johnson syndrome and toxic epidermal necrolysis

Ophthalmic manifestations of the acute stages of Stevens-Johnson syndrome (SJS) and toxic epidermal necrolysis (TEN) range from conjunctival hyperemia to near-complete sloughing of palpebral conjunctiva and lid margins.^[159]^ Acute ocular involvement is reported to occur in up to 88% of the cases.^[159]^ It remains unclear whether the severity of ocular involvement is any different between SJS and TEN.^[160]^ Long-term adverse consequences following the acute stage of ocular surface disease include severe dry eyes, symblepharon formation, corneal limbal stem cell deficiency, and corneal scarring.^[160]^

##  Toxic conjunctivitis

It has been recently realized that long-term use of topical eye medications may induce ocular surface changes including dry eyes, conjunctival inflammation, ocular surface fibrosis, and scarring.^[161,162]^ Another area where the side effects of topical eye drops cause significant ocular morbidity is their use in glaucoma and in patients who have undergone glaucoma surgery. Subclinical infiltration of the conjunctival epithelium and substantia propria by inflammatory cells has also been reported.^[163,164]^ The published literature during the past decade has pointed to the deleterious effects of benzalkonium chloride (BAK), which is used as a preservative in eye drops, on the ocular surface.^[165]^

Allergic reactions are the most clinically noticeable side effect of the eye drops; however, they are far less frequent and harmful than their adverse toxic side effects.^[166]^ The allergic reaction to eye drops includes simple conjunctival congestion, papillary conjunctivitis, and GPC.^[165]^ The signs and symptoms usually manifest a few days after starting the offending eye drop and tend to resolve quickly when the medication is stopped.^[166]^

Observational studies have confirmed the high prevalence of dry eyes in glaucoma patients related to the number of eye drops being used. This ranges from 11% in those who use only one eye drop to 43% in those who use two or three different eye drops.^[167]^ Similarly, a cross-sectional study evaluating the ocular surface in 101 patients being treated for glaucoma reported that approximately 60% of them were symptomatic in at least one eye.^[168]^ In a survey performed on 300 patients in the US between 2001 and 2004, adverse side effects were reported to be the second most common reason for switching eye drops.^[169]^

Increase in fibroblast density in the conjunctiva, and development of subconjunctival fibrosis has been reported in patients who use antiglaucoma drops chronically.^[165]^ In a series of 145 patients, Thorne et al reported that exposure to antiglaucoma eye drops was the primary reason for development of pseudopemphigoid.^[170]^

Despite the indisputable data and the findings from multiple observational studies on the harmful side effects of BAK, it is still used as the main preservative ingredient in most eye drop preparations due to lack of a better alternative.^[165]^ Limiting the exposure to preservatives may diminish the toxic side effects of eye drops; this will likely lead to higher patient compliance and result in a more favorable clinical outcome, especially in those who need to be on antiglaucoma medications.

##  Summary

Approximately 1% of all patient visits to their primary care physician is conjunctivitis related, and the estimated cost of infectious conjunctivitis to the healthcare is more than $800 million annually in the US alone.^[2]^ The first step in approaching a patient with presumed conjunctivitis is to rule out serious ocular conditions that present with “red eye”, mimicking conjunctivitis. This must be done with obtaining a thorough history and performing a detailed ophthalmologic and physical examination. Ancillary laboratory testing and imaging are also important components of evaluating these patients. Various studies have demonstrated that obtaining a thorough history is essential to narrow down the differential diagnosis and discover the underlying etiology for the conjunctivitis, while relying solely on the presenting signs and symptoms can be misleading and often leads to an inaccurate diagnosis. Viral conjunctivitis followed by bacterial conjunctivitis are the most common causes of infectious conjunctivitis.^[15,25,81]^ The majority of viral conjunctivitis cases are due to adenoviruses,^[28]^ and the use of rapid antigen test to diagnose adenoviral conjunctivitis may present an appropriate strategy to avoid overuse of antibiotics. Bacterial pathogens are isolated in half of the cases of conjunctivitis,^[61]^ and approximately 60% of culture-positive cases are known to be self-limited.^[80]^ Cultures should be obtained from the conjunctival swabs of patients that do not respond to therapy, and those suspected to have chlamydial infection and hyperacute conjunctivitis.^[47]^ Treatment with topical antibiotics is usually recommended for suspected cases of chlamydial and gonococcal conjunctivitis and contact lens wearers.^[61,80]^ The majority of cases of allergic conjunctivitis are due to seasonal allergies. Antihistamines and mast cell stabilizers are widely used for treating allergic conjunctivitis. Steroids must be used judiciously and only when indicated. For patients with chronic conjunctivitis, possibility of systemic diseases and adverse effects of eye drops with preservatives should be kept in mind.
